# Automated classification of mandibular canal in relation to third molar using CBCT images

**DOI:** 10.12688/f1000research.154985.1

**Published:** 2024-09-02

**Authors:** Neil Abraham Barnes, Winniecia Dkhar, Sharath S, Yogesh Chhaparwal, Veena Mayya, Roopitha C H

**Affiliations:** 1 Department of Medical Imaging Technology, Manipal College of Health Professions, Manipal Academy of Higher Education, Manipal, Karnataka, 576104, India; 2 Department of Oral Medicine and Radiology, Manipal College of Dental Sciences, Manipal Academy of Higher Education, Manipal, Karnataka, 576104, India; 3 Departments of Information & Communication Technology, Manipal Institute of Technology, Manipal Academy of Higher Education, Manipal, Karnataka, 576104, India

**Keywords:** Artificial Intelligence; CBCT; CNN; Mandibular Canal; Machine Learning; Third Molar

## Abstract

**Background:**

Dental radiology has significantly benefited from cone-beam computed tomography (CBCT) because of its compact size and low radiation exposure. Canal tracking is an important application of CBCT for determining the relationship between the inferior alveolar nerve and third molar. Usually, canal tacking is performed manually, which takes a lot of time. This study aimed to develop an artificial intelligence (AI) model to automate classification of the mandibular canal in relation to the third molar.

**Methods:**

This retrospective study was conducted using 434 CBCT images. 3D slicer software was used to annotate and classify the data into lingual, buccal, and inferior categories. Two convolution neural network models, AlexNet and ResNet50, were developed to classify this relationship. The study included 262 images for training and 172 images for testing, with the model performance evaluated by sensitivity, precision, and F1 score.

**Results:**

The performance of the two models was evaluated using a 3 × 3 confusion matrix, with the data categorized into 3 clases: lingual, buccal, and inferior. The mandibular canal and third molar have a close anatomical relationship, highlighting the need for precise imaging in dental and surgical settings. To accurately classify the mandibular canal in relation to the third molar, both AlexNet and ResNet50 demonstrated high accuracy, with F1 scores ranging from 0.64 to 0.92 for different classes, with accuracy of 81% and 83%, respectively, for accurately classifying the mandibular canal in relation to the third molar.

**Conclusion:**

The present study developed and evaluated AI models to accurately classify and establish the relationship between the mandibular canal and third molars using CBCT images with a higher accuracy rate.

## Introduction

Cone-beam computed tomography (CBCT) has revolutionized oral and maxillofacial radiology. Owing to its compact size, low cost, and low ionizing radiation exposure, CBCT has rapidly become the preferred imaging modality for dental radiology. ^1^ In CBCT, a cone-shaped beam scans the patient in 360-degree rotation and acquires a large field of view (FOV). ^2^ CBCT has played a major role in dental imaging owing to its various clinical applications such as orthodontics, temporomandibular joint testing, endodontics, periodontics, implantology, and mandibular canal tracking. ^3^^,^^4^

Mandibular canal tracking is an application of CBCT imaging that is widely used to track the extension of the mandibular canal, which houses the inferior alveolar nerve (IAN). The alveolar nerve, with an average length of 2.00 mm to 5.00 mm, starts from the mandibular foramen and runs through the mental foramen. ^5^ The IAN plays a significant role in facial motor and sensory functions. The mandibular nerve innervates the masticatory muscles of smaller muscles and provides sensory input from the lower face. Damage to the mandibular nerve can lead to persistent or worsening neurosensory deficits including numbness, pain, and loss of taste in the lips, mucosa, and tongue. It can cause pain in the lower teeth, lower jaw, and lower lips when the IAN is dysfunctional, thereby affecting speech and chewing.

As CBCT provides a three-dimensional view of the structures involved, it has become a crucial tool for evaluating the anatomical relationship between the nerve canal and third molar roots. Radiographic assessment is necessary to determine this relationship. ^6^ Extracting an impacted third molar is a common oral and maxillofacial surgery procedure, but it can result in postoperative complications and IAN injury. ^7^

Hence, tracking the canal using CBCT and establishing the relationship between the third molar and mandibular canal is very important because the third molar is the most impacted tooth in the human dental anatomy. ^8^^,^^9^ Currently, classification is performed manually, which is less accurate and more time-consuming. Artificial intelligence (AI) has shown significant success in various medical imaging fields, including dentistry. AI is a component of computer science that deals with creating an intelligent computer system that displays traits associated with intelligence in comprehending human behavior, language, education, logic, problem-solving, and many more. ^10^^,^^11^^,^^12^ Convolutional neural network (CNN) AI techniques have become dominant in various computer vision tasks and have been applied in fields such as radiology for image analysis. Manual tracing and establishing the canal relationship can be performed automatically, more accurately, and faster using a CNN. The present study aimed to develop an AI model to establish the relationship between the third molar and the mandibular canal using CBCT images.

## Methods

This is a retrospective study. Ethical approval was obtained from the Institutional ethical committee (IEC2:322/2022) of Kasturba Medical College and Hospital, Manipal, India on 14th July 2022.CBCT images of the mandible were collected from Department of Oral Medicine and Radiology, Manipal College of Dental Sciences, which were acquired using an i-CAT 17-19 Platinum (Imaging Sciences International LLC, USA) CBCT scanner. The scan parameters were in the range of 85-90 kVp, 6mA, as specified by the vendor. The inclusion criteria were that the CBCT images collected should contain a bilateral third molar tooth and an age range of 18-60 years. The exclusion criteria were the presence of a dental prosthesis and missing third molar. A total of 580 studies were screened based on the inclusion and exclusion criteria and 434 CBCT images were included.

### Data annotation

**Figure 1.**
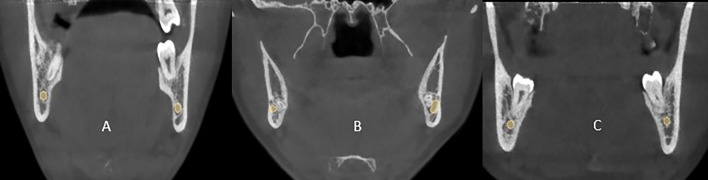
(A) CBCT image of a mandibular canal passing through the lingual side of third molar. (B) The mandibular canal passes through buccal side of third molar. (C) Canal passing through inferior side of third molar.

CBCT images were manually classified into three Categories, Lingual, Buccal, and Inferior, by locating the canal in relation to the third molar using 3D Slicer an open source software, ^13^ as shown in Figure 1.

### Classification of the data

To develop and test a machine learning model, 434 images were divided into two subsets, including 262 images for training and 172 for testing the model. Two models, AlexNet and ResNet were developed based on convolutional neural networks (CNN).

### Machine learning models

**Model 1: -** AlexNet

**Figure 2.**
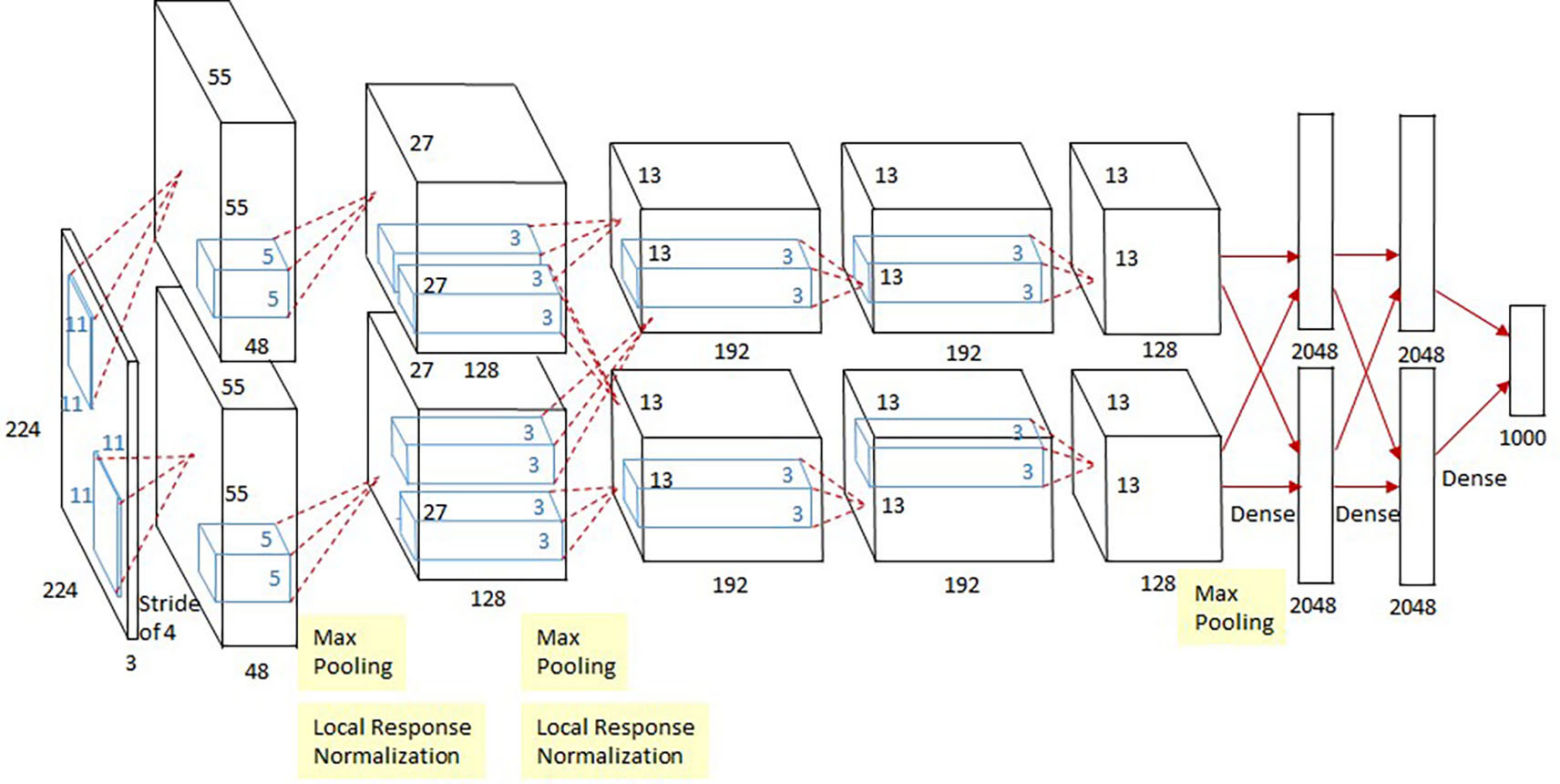
An architecture of AlexNet.

The AlexNet architecture is a classic convolutional neural network (CNN) that consists of convolution, max pooling, and dense layers. Eight layers were present: five convolutional layers, three max-pooling layers, and three fully connected layers. There were two copies of the network, each running on its own GPU. The AlexNet architecture is shown in Figure 2.

**Model 2: - ResNet50**

**Figure 3.**
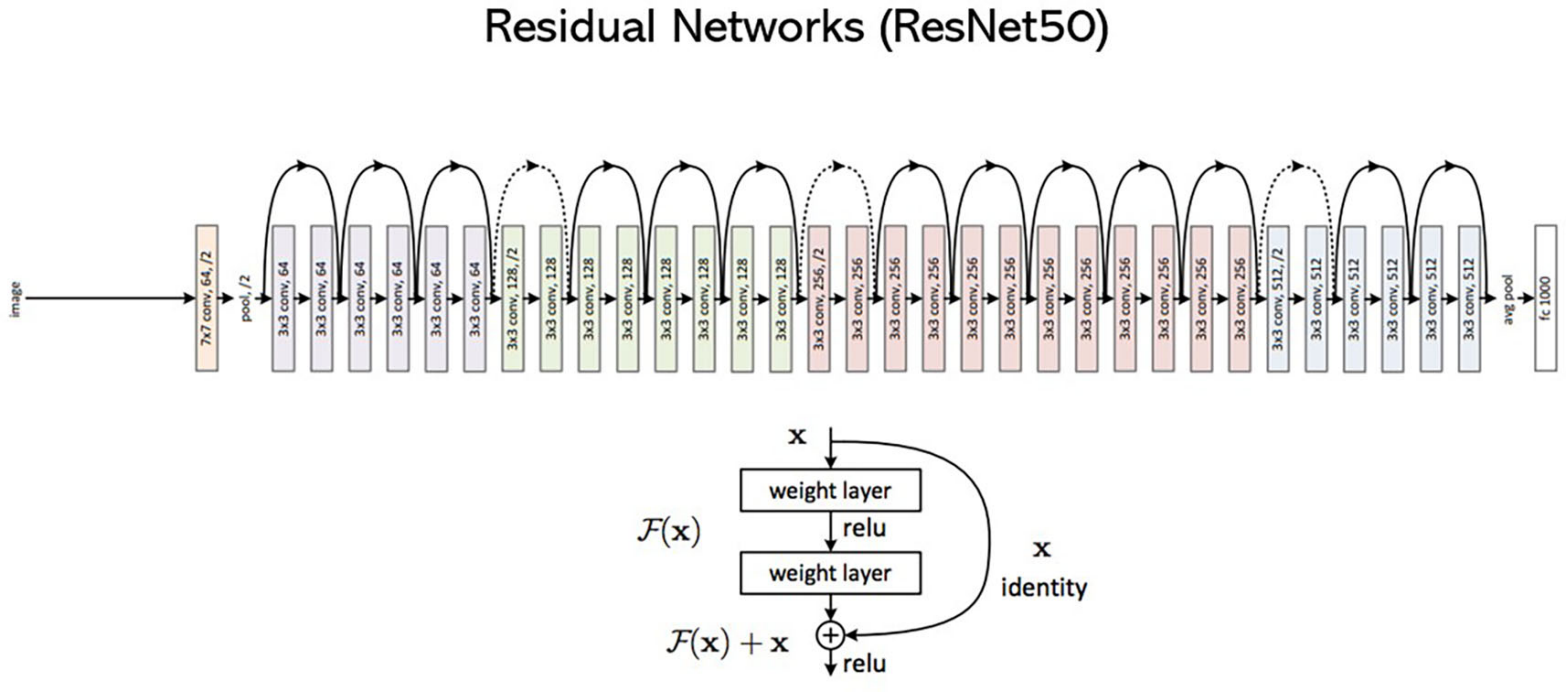
An architecture of ResNet50.

ResNet, which stands out as the Residual Network, is a type of CNN. It is a 50-layer CNN (48CNN layer, 1 MaxPool layer, and 1 Average pool layer). The networks were formed by stacking the residual blocks. It is comprised of 34 weighted layers. 3.6 billion FLOPs can be achieved with their implementation, while 1.8 billion FLOPs can be achieved with a smaller 18-layer ResNet. In addition, if the size of the feature maps is halved, the number of filters is doubled to preserve the complexity of the time of each layer. Owing to its 50-layer design, ResNet employs a bottleneck design for building blocks. Figure 3 shows the architecture of the ResNet.

## Results

The mandibular canal and third molar have a close anatomical relationship that is essential in dental and surgical contexts. Cone-beam computed tomography (CBCT) and panoramic radiography have been used in several studies to study this relationship. As the inferior alveolar nerve (IAN) passes through the mandibular canal, its position is anatomically important. It has been shown that the mandibular canal has a close relationship with a high percentage of third molars, and this relationship is important to consider in dental imaging because damage to the IAN can occur during surgery on mandibular third molars. Therefore, locating the mandibular canal is a vital part of dental imaging. In the present study, we developed an AI model to automatically establish the relationship between the mandibular canal and the third molar.

In the present study, 172 data points were used to test the model. Two model sets were developed: AlexNet and ReSNet50, which are based on a CNN. The models were then evaluated for their performance. The performance of these models was assessed using a 3 × 3 confusion matrix with output comprising true positive (TP), True Negative (TN), and misclassified cases (MC), in which data are categorized into three classes: lingually (A), buccal (B), and inferior (C). The results of the confusion matrix for AlexNet and ResNet are listed in Tables 1 and 2, respectively.

**Table 1.** Confusion matrix showing the classification results of the AlexNet model.

	TP	TN	MC
**Lingual (A)**	125	3	3
**Buccal (B)**	5	8	1
**Inferior (C)**	20	0	7

* TP-True Positive, TN-True Negative, MC-Miss Classified.

**Table 2.** Confusion matrix showing the classification results of the ResNet50 model.

	TP	TN	MC
**Lingual (A)**	121	3	7
**Buccal (B)**	5	6	3
**Inferior (C)**	10	2	15

The accuracy and performance of the models to classify the relationship between the third molar was evaluated using its Precision, Sensitivity/Recall, and F1 Score. The AlexNet model yielded an F1score of 0.89 for Class A (Lingual), 0.64 for Class B (Buccal), and 0.37 for Cass C (Inferior **),** with an overall accuracy of 81%, as shown in Table 3 & Table 4.

**Table 3.** Performance result of AlexNet model.

Categories	Precision	Sensitivity/Recall	F1- Score	Support
**Lingual (A)**	0.83	0.95	0.89	131
**Buccal (B)**	0.73	0.57	0.64	14
**Inferior (C)**	0.64	0.26	0.37	27

**Table 4.** Accuracy of the AlexNet model.

Classification Report	Precision	Recall	F1-Score	Support
**Accuracy**			0.81	172
**Macro average**	0.73	0.59	0.63	172
**Weighted average**	0.79	0.81	0.79	172

The ResNet model yielded an F1score of 0.92, 0.43, and 0.5% for Class A (Lingual), Class B (Buccal), and 0.56 for Class C (inferior **),** with an overall accuracy of 83% for accurately classifying the mandibular canal in relation to the third molar, as shown in Tables 5 and 6.

**Table 5.** Performance result of ResNet50 model.

Categories	Precision	Sensitivity/Recall	F1- Score	Support
**Lingual (A)**	0.89	0.92	0.91	131
**Buccal (B)**	0.55	0.43	0.48	14
**Inferior (C)**	0.60	0.56	0.58	27

**Table 6.** Accuracy of ResNet50 model.

Classification Report	Precision	Recall	F1-Score	Support
**Accuracy**			0.83	172
**Macro average**	0.68	0.64	0.65	172
**Weighted average**	0.82	0.83	0.82	172

## Discussion

Machine learning has been used in dental imaging for various purposes such as the diagnosis and treatment of oral diseases. Deep learning in dental imaging has been successful in detecting caries, periodontal bone loss, and other dental problems. Mandibular canal and third molar relationships have been established in dentistry using machine-learning models. Deep learning models have been used to analyze panoramic radiography and other imaging techniques to determine the relationship between mandibular third molars and the mandibular canal. Dental imaging involves identifying the exact location of the mandibular canal in relation to the third molar to prevent complications during surgery.

In the present study, we developed two machine learning models, AlexNet and ResNet50, for classifying the mandibular canal in relation to the third molar into three different classes based on their location: lingual, buccal, and inferiorly. Various studies have been conducted with different machine learning algorithms using panoramic radiographs to establish the relationship between the third molar and mandibular canal. Few studies have been conducted on CBCT images. We developed and evaluated two machine learning models based on CNN using CBCT images.

The performance of the CNN models was evaluated using a confusion matrix in which the AlexNet model identified 125 images as TP, three images as TN, and misclassified three images as Class A out of 131 lingual data similarly for Class B out of 14 images; the model identified five images as TP, eight as TN, and misclassified one image, and for the data sets involving Class C AlexNet model, we identified 20 images accurately and misclassified seven out of 27 images. Similarly, the ResNet model identified 121 images as TP, three images as TN, and misclassified 7 images as Class A out of 131 lingual data, also for Class B, five images were identified as TP, six as TN and misclassified 3 images and for the datasets involving Class C, the AlexNet model identified 20 images accurately and misclassified 7 images

Recently, various studies have reported the application of CNN with promising results in the staging of tooth development, detection, and classification of third molars and canals. Artificial neural networks with deep feed-forward architectures are used for a wide range of tasks such as recognizing activities, recognizing text, recognizing faces, describing images, detecting objects, and localizing them. Because of the ability of this technique to learn abstract features from spatial data, it can be used to efficiently classify images. It has multiple architectures, including ResNet and AlexNet. ^14^ AlexNet are two popular deep-learning models used in dental imaging applications. According to the size and complexity of the dataset, as well as the specific characteristics of the dental images, the ResNet and AlexNet models can perform differently in dental imaging.

The AlexNet model proposed by Krizhevsky et al. (2012 ^15^) is considered a pioneer in deep learning for image classification because it is relatively shallow. This algorithm has eight layers, including three fully connected and five convolutional layers. For enhanced generalization, local response normalization and dropout are incorporated into the architecture; if the dataset is not extensive and a simpler model is preferred, the relatively shallow architecture might be advantageous in dental imaging. The introduction of ResNet by He et al. in 2015 ^16^ was an important breakthrough in deep learning. The most innovative feature of ResNet is that it consists of residual blocks, which contain skip connections that allow information to flow directly from one layer to another. Consequently, the vanishing gradient problem is mitigated, and extremely deep networks can be trained more easily.

Several studies have tested the performance of ResNet and Alex Net in dental imaging by using them for different tasks. A study conducted by Mu-Qing Liu et al., ^17^ developed a U-Net model for detecting and segmenting the mandibular canal, while the ResNet-34 model was used to classify the relationship between the mandibular canal and third molars. The classification model had 90.2% sensitivity, 95.0% specificity, and 93.3% accuracy. In the present study, we developed two CNN models: model 1 was 81% accurate, and model 2 was 83% accurate in classifying the mandibular canal as related to the third molar.

^18^Using a deep learning model, Sukegawa et al. studied how the inferior alveolar nerve is related to the mandibular third molar on panoramic radiography. To classify the positional relationship between the two structures, CNN models were used, including ResNet50 and ResNet50v2, in which ResNet50v2 was the top performer in both the continuity analysis and contact analysis using the SAM optimizer. For continuity analysis, the accuracy and AUC were 0.766 and 0.843, respectively. A study proposed the use of ResNet-152 and AlexNet to detect caries and non-carious teeth in panoramic dental images, which had an accuracy rate of 96.08%. ^19^ Similarly, Katsuki Takebe et al. ^20^ performed a study in which the authors developed a YOLO model for evaluation of contact between the third molar and alveolar nerve automatically resulting in a f1 score of 0.908, while the AlexNet and ResNet models in the present study showed f1 score of 0.81and 0.83, respectively, in classifying the mandibular canal in relation to the third molar.

In CBCT, tracing the canals manually and establishing their relationship to the third molars is performed by a skilled operator meticulously tracing the root canals. It is widely used and accepted owing to its accuracy and reliability. Despite this, the process is not only time-consuming but also requires expertise and may be subject to variability within and among operators. To accurately trace the canal pathways, the operator relies on the knowledge and experience of dental anatomy. Despite its high level of precision, manual segmentation is limited when dealing with complex anatomies or large datasets. Automatic canal segmentation methods have gained popularity with the advent of artificial intelligence (AI) and machine learning. Using these techniques, root canals can be identified and delineated automatically, without the need for manual intervention. Its primary objective is to improve efficiency and reduce operator dependency, while providing consistent results across datasets. With AI systems, patients can obtain optimal imaging based on their clinical needs, presentation, and region of interest. They can also detect artifacts or distortions that interfere with interpretation. ^21^^,^^22^ The radiation dose can also be reduced by maintaining good image quality, as has been reported in previous studies, ^23^^,^^24^ to keep radiation doses within the threshold limit for all radiosensitive organs, while maintaining good image quality.

Based on the results of this study, it is noted that AI algorithms based on CNN can be used as a better alternative method with great accuracy for identifying the mandibular canal and establishing its relation to the third molar in CBCT, which can help reduce the manual method. The application of AI-based algorithms can help dental professionals diagnose and assess the root anatomy in relation to the third molar. This will enable them to provide appropriate treatment without damaging the inferior alveolar nerve or causing any complications. Overall, AI technology has the potential to revolutionize the field of dentistry and change the way dental professionals diagnose and treat various diseases.

Although the models developed in the present study have demonstrated high accuracy and precision with the inclusion of two different models, which allowed a comprehensive assessment of the performance of these architectures in dental imaging, there are some limitations in the present study: the age range was restricted to 18-60 years which might limit the generalizability of the results. In addition, CBCT images were collected from a single facility, which may limit the diversity of cases and anatomical variations in the dataset.

## Conclusion

In conclusion, this study developed and evaluated two CNN-based models, AlexNet and ResNet50, for classifying the relationship between third molars and the mandibular canal using CBCT images. Both models demonstrated high accuracy and F1 scores across different classes, indicating better performance. These AI-based models have the potential to enhance the diagnostic efficiency and improve patient care when integrated into routine clinical practice.

### Ethics and consent

This is retrospective study. Ethical approval was obtained from the Institutional ethical committee (IEC2:322/2022) of Kasturba Medical College and Hospital, Manipal India on 14 ^th^ July 2022. No consent was obtained in accordance with the ethical approval, as this is a retrospective study.

## Data Availability

Figshare: Annotated data set, https://doi.org/10.6084/m9.figshare.26502916.v1. ^25^ This project contains the following underlying data: The annotated data sets of mandibular canal classified as Lingual, Buccal and Inferior Data are available under the terms of the Creative Commons Attribution 4.0 International license (CC-BY 4.0).
