# Supplementary material for: Automated classification of mandibular canal in relation to third molar using CBCT images
Source: F1000Res. 2024 Sep 2;13:995. [Version 1] doi: 10.12688/f1000research.154985.1 (PMC11803391; doi:10.12688/f1000research.154985.1)
Supplement: Peer review history

# Reviewer report: Reviewer response for version 1

**DOI:** 10.5256/f1000research.170094.r345485

**Published:** 2024-12-27

**Authors:** Antonio Lo Casto

**Affiliations:** 1 University of Palermo, Palermo, Sicily, Italy

The paper is an interesting contribution on the use of CNN to classify the relationship between third molar and mandibular canal.

 Just some minor review should be made.

 1. The length of the alveolar nerve is longer than what reported (2.00 mm to 5.00 mm) in the section "Introduction".

 2. The first part of the section "Results" from "The mandibular canal... to ...and the third molar" is more suited for the sections "Introduction" or "Discussion".

Is the work clearly and accurately presented and does it cite the current literature?

Partly

If applicable, is the statistical analysis and its interpretation appropriate?

Yes

Are all the source data underlying the results available to ensure full reproducibility?

Yes

Is the study design appropriate and is the work technically sound?

Yes

Are the conclusions drawn adequately supported by the results?

Yes

Are sufficient details of methods and analysis provided to allow replication by others?

Yes

Reviewer Expertise:

Radiology and Imaging.

I confirm that I have read this submission and believe that I have an appropriate level of expertise to confirm that it is of an acceptable scientific standard.

## References

[1] : Artificial Intelligence for Classifying the Relationship between Impacted Third Molar and Mandibular Canal on Panoramic Radiographs. *Life (Basel)* .2023;13(7) : 10.3390/life13071441 10.3390/life13071441 37511816 PMC10381483

# Reviewer report: Reviewer response for version 1

**DOI:** 10.5256/f1000research.170094.r332755

**Published:** 2024-10-21

**Authors:** Dr. Rohit Gadda

**Affiliations:** 1 Mahatma Gandhi Mission Dental College and Hospital, Navi Mumbai, India

This is an interesting manuscript investigating automated classification of mandibular canal in relation to third molar using CBCT images. Manuscript is well written. However it is recommended to authors to describe detailed protocol for selection of CBCT images (coronal section and level of section) collected as CBCT scan is 3 dimensional dataset with multiple 2 dimensional images in different planes can be created. Figure 1. (A) shows mandibular canal passing inferior to the  third molar. So authors are requested to revisit this figure.

 In discussion section, 8th paragraph 'In CBCT, tracing of the canals...' context of root canal or canals is to be revised and rewritten.

Is the work clearly and accurately presented and does it cite the current literature?

Yes

If applicable, is the statistical analysis and its interpretation appropriate?

Yes

Are all the source data underlying the results available to ensure full reproducibility?

Yes

Is the study design appropriate and is the work technically sound?

Yes

Are the conclusions drawn adequately supported by the results?

Yes

Are sufficient details of methods and analysis provided to allow replication by others?

Yes

Reviewer Expertise:

Cone Beam Computed Tomography (CBCT), Oral and Maxillofacial imaging, Oral and Maxillofacial diseases.

I confirm that I have read this submission and believe that I have an appropriate level of expertise to confirm that it is of an acceptable scientific standard.
